# Botulinum Neurotoxins (BoNTs)—Antibody and Vaccine

**DOI:** 10.3390/toxins10120495

**Published:** 2018-11-26

**Authors:** Jianlong Lou, James D. Marks

**Affiliations:** Department of Anesthesia and Perioperative Care, University of California, San Francisco, Zuckerberg San Francisco General Hospital and Trauma Center, Room 3C-38, 1001 Potrero Avenue, San Francisco, CA 94110, USA

Botulism, caused by exposure to one or more of the eight serotypes of botulinum neurotoxins (BoNTs) (BoNT/A through H), is often fatal without rapid treatment. Botulism occurs sporadically all over the world, due to environmental exposure, contaminated food, wound infection, or overdose from genuine or counterfeit BoNT-based drug products.

In the past few decades, cosmetic and medical uses for an ever-expanding list of disease indications, from depression to spasticity [1], for BOTOX^®^ [2] and other [3] approved BoNT-based drugs, such as Myobloc^®^, Xeomin^®^, and others, has resulted in an explosive growth in both the availability of BoNT and number of persons treated. Furthermore, intentional misuse of BoNTs by bioterrorists [4] or rogue nations [5] remains a risk. With increasing BoNT production, and use and mis-use, comes the need for treatment and prevention of BoNT intoxication.

In this special issue of “Botulinum Neurotoxins (BoNTs)—Antibody and Vaccine”, two review articles and ten original research papers on medical countermeasures for botulism, and therapeutic use of a new BoNT/A-based drug, are reported. Briefly, Grace Sundeen and Joseph T. Barbieri [6] reviewed the current status of DNA-based, viral vector-based, and recombinant protein-based vaccines against botulism. A vaccine is needed since the pentavalent toxoid vaccine is no longer available. Christine Rasett-Escargueil, Arnaud Avril, and their colleagues [7] reviewed the status of the EU Framework program for developing humanized AntibotABE antibodies. In this issue, we, along with our collaborators at UCSF, report engineering of a tri-epitopic antibody that recapitulates the neutralizing activity of a combination of three antibodies to BoNT/A, potentially offering a simpler route to an antibody-based therapeutic [8]. Along with Consuelo Garcia-Rodriguez, we also report development of a three-antibody combination that potently neutralizes BoNT/E [9], analogous to that reported for BoNT/A and/F. Yagmur Derman, Katja Selby, and their collaborators across the EU report on a humanized scFv-FC fusion, derived from immunized macaques and its potential in the neutralization of BoNT/E [10]. Osnat Rosen, Amram Torgeman, Ran Zichel, and their team from Israel, reported their development of an in vitro potency assay for selected anti-BoNT antibodies [11] and their research on the role of homologous Fc fragment in the potency and efficacy of anti-BoNT antibody [12]. Fetweh H. Al-Saleem, Rashmi Sharma, Scott K. Dessain, and their co-workers from the United States of America, presented the development of a fusion protein for red blood cell adherence of immune complexes containing BoNT, to improve neutralization and macrophage uptake [13]. Nicola Bak, Dorothea Sesardic, and their team from the United Kingdom, reported on the application of SiMa cells for cell-based neutralization test for BoNT/A and BoNT/E [14]. On the vaccine development side, Robert P. Webb, Theresa J. Smith, and their team from USAMRIID, reported on the production of non-toxic holoproteins and recombinant BoNT toxin domain subunits as vaccine candidates against multiple serotypes [15], Denis Y. Otaka, Felipe M. Salvarani, and their team from Brazil, reported on the humoral response of buffalos to a recombinant vaccine against BoNT/C and D [16]. Finally, Hyun Jung Chang, Bo Young Hong, Jeong-Yi Kwon, and their teams from South Korea, report results from a clinical trial on the efficacy and safety of Botulax for the treatment of dynamic equinus foot deformity in children with cerebral palsy [17].

We hope the papers presented here will provide new insight into this intriguing protein, and prevention or treatment of botulism. We appreciate the contributions of BoNT researchers from around the world to this special issue.
